# Barriers to delivering trauma‐focused interventions for people with psychosis and post‐traumatic stress disorder: A qualitative study of health care professionals’ views

**DOI:** 10.1111/papt.12387

**Published:** 2022-02-05

**Authors:** Eleanor Chadwick, Jo Billings

**Affiliations:** ^1^ Department of Clinical, Educational and Health Psychology UCL London UK; ^2^ Division of Psychiatry UCL London UK

**Keywords:** barriers, post‐traumatic stress disorder, psychosis, trauma, trauma‐focused therapy, treatment

## Abstract

**Objectives:**

Trauma‐focused interventions have been shown to be effective treatments for post‐traumatic stress disorder (PTSD), and clinical guidelines support their use with people with psychosis. Despite this, they are used relatively infrequently in this population. We sought to explore UK health care professionals’ perceptions of what impedes or facilitates the use of trauma‐focused interventions among people with psychosis and PTSD.

**Design:**

A qualitative study using constructivist grounded theory methodology.

**Methods:**

We conducted semi‐structured interviews with 18 health care professionals working within the commissioning and delivery of clinical services for people with psychosis.

**Results:**

Three inter‐related barriers to the use of trauma‐focused interventions were conceptualized: coherent understanding; structural support; and safe space.

**Conclusions:**

Delivery of trauma‐focused interventions within routine clinical practice may be supported by attention to the coherent integration of discussion of trauma into the clinical discourse of services; the processes, pathways, and organizational culture that facilitate access to treatment; and training that targets clinician confidence and skills.

**Practitioner points**

- Health care professionals describe multiple barriers to the delivery of trauma‐focused interventions for people with psychosis and PTSD: as a result, providing these interventions continues to be considered the exception rather than the rule.
- Findings highlight perceived barriers in the form of coherent integration of the discussion of trauma into the clinical discourse of psychosis services; the structural support for the delivery of trauma‐focused interventions; and a safe space for intervention to occur.
- A range of potential opportunities to improve the delivery of trauma‐focused interventions are identified including the use of psychosocial team formulation sessions, organizational interventions, and training focused on the development of staff confidence and skills.

## INTRODUCTION

The association between trauma and psychosis is well‐established. Individuals with psychosis are consistently shown to have experienced high levels of trauma (de Bont et al., 2015; Gibson et al., 2016; Varese et al., 2012), and have an increased risk of continued exposure to traumatic events including experiences of psychosis, psychiatric treatment, hospitalization (Berry et al., 2013), and victimization (Maniglio, 2009).

High exposure to traumatic events places people with psychosis at an increased risk of developing post‐traumatic stress disorder (PTSD; Grubaugh et al., 2011). Meta‐analyses estimate the prevalence of PTSD in people with psychosis at 12.4% (Achim et al., 2011): however, this may underestimate the true prevalence, given findings from a recent review which placed the prevalence of psychosis‐related PTSD between 14% and 47% (Buswell et al., 2021). Comorbid diagnoses of psychosis and PTSD are associated with higher symptom levels, poorer social functioning and quality of life (Grubaugh et al., 2011; Mueser et al., 2010), as well as with higher use of and worse outcomes from health care (Insel, 2008; Switzer et al., 1999).

Treatment of PTSD in people with psychosis has increasingly been recognized as a clinical priority. Clinical guidelines recommend that individuals presenting with first‐episode psychosis should be assessed and offered treatment for PTSD (National Institute for Health and Care Excellence (NICE), 2014). Despite this, there is no formal recommendation for assessment and treatment of PTSD outside of first‐episode psychosis in current UK guidance.

### Trauma‐focused interventions

Trauma‐focused psychological interventions including cognitive behavioural therapy (tf‐CBT) or eye‐movement desensitisation and reprocessing (EMDR) are recommended first‐line interventions for PTSD (American Psychological Association, 2017; NICE, 2018). These interventions should be distinguished from trauma‐informed approaches which refer to broader organizational awareness of, and attendance to, the prevalence of and diverse reactions to traumatic experiences. Trauma‐informed approaches have been written about extensively elsewhere (Reeves, 2015; Sweeney et al., 2016).

Large meta‐analyses of tf‐CBT and EMDR demonstrate reductions in both symptoms and distress in individuals with a diagnosis of PTSD (Lewis et al., 2020; Mavranezouli et al., 2020). However, people with psychosis are typically excluded from research trials to minimize sample heterogeneity and due to harm expectancies in this population (Swan et al., 2017). As a result, the efficacy of interventions for PTSD for people with psychosis is uncertain. NICE guidelines for the treatment of psychosis (2014) identify no contraindication for trauma‐focused interventions and classify further research in this area as a key priority.

An emerging evidence‐base offers tentative support for the use of trauma‐focused interventions for people with psychosis and PTSD. There is promising evidence from randomized controlled trials which support the efficacy (Mueser et al., 2008, 2015; van den Berg et al., 2015) and safety (van den Berg, de Bont, et al., 2016; van den Berg, van der Vleugel, et al., 2016) of trauma‐focused interventions for the treatment of PTSD in this group. While meta‐analyses report mixed interpretations of the available data (Brand et al., 2018; Sin & Spain, 2017; Swan et al., 2017), discrepancies may be attributable to differences in analytic strategies and heterogeneity in the interventions and participant samples across trials.

Despite the growing clinical interest, emerging research remains tentative regarding the efficacy of trauma‐focused interventions with people with psychosis, and further research is clearly needed. Harm expectancies, including the fear of symptom exacerbation, destabilizing the patient and adverse events (van den Berg, de Bont, et al., 2016; van den Berg, van der Vleugel, et al., 2016), may contribute to reluctance by ethical boards to support the conduct of needed trials.

### Clinical practice

Despite the prevalence of PTSD in people experiencing psychosis, PTSD in this population is frequently under‐recognized in clinical services (de Bont et al., 2015; Lommen & Restifo, 2009). Even when PTSD is identified, individuals are rarely offered trauma‐focused treatment (Becker et al., 2004). Understanding this discrepancy is important to reduce the potentially detrimental consequences of failure to identify and treat PTSD in this population (Álvarez et al., 2012).

Previous research has explored barriers and facilitators to the implementation of trauma‐focused interventions with people with PTSD more broadly. In a recent systematic review, Finch et al., (2020) synthesized findings from 34 published studies examining barriers and facilitators to providing trauma‐focused interventions. They identified four levels of barriers and facilitators covering intervention, client, clinician, and system factors. The most commonly cited barriers identified included inflexibility of manualized approaches, fear of increasing client distress, working with comorbidities, and a lack of training and support.

Little research has directly examined the barriers specific to the delivery of trauma‐focused treatment with people with psychosis. Clinician attitudes, knowledge, and self‐efficacy in delivering treatment have been shown to be predictive of the adoption of interventions for other psychological difficulties (Harned et al., 2013; Salyers et al., 2004). The perspectives of health care professionals may therefore offer important insights into the potential barriers to the delivery of trauma‐focused interventions with people with psychosis.

Two studies have previously explored the perspectives of American clinicians towards trauma‐focused interventions for people with Severe Mental Illness (Frueh et al., 2006; Salyers et al., 2004): these studies describe significant client‐ and clinician‐related barriers to their use with this population. Client‐related barriers included symptoms interfering with treatment; client unwillingness; cognitive impairment; and communication difficulties (Salyers et al., 2004). Clinician‐related barriers to treatment included clinician anxiety (Frueh et al., 2006); lack of knowledge and experience; as well as staff perceptions regarding their competence and confidence delivering interventions; the usefulness of interventions; and agency support (Salyers et al., 2004). Clinicians’ attitudes to treatment, in particular, were predictive of whether clinicians had assessed or treated PTSD with clients (Salyers et al., 2004). Severe mental illness describes a heterogeneous group and it is hard to assess which of these barriers apply specifically to treatment for people with psychosis. Further, given the rapid development of research and clinical guidelines in this area, barriers to treatment may have shifted significantly since these studies were conducted.

One study has previously examined the perspectives of Australian clinicians towards trauma‐focused interventions for people with first‐episode psychosis (Gairns et al., 2015). The mixed‐methods design synthesized quantitative and qualitative data from questionnaires with qualitative data from focus groups. Additional barriers to the delivery of trauma‐focused interventions to people with psychosis included perceived mental health risks to clients, workload pressures, and poor client engagement (Gairns et al., 2015). The mixed methodology highlighted apparent contradictions in staff reports that are hard to interpret. Notably, despite 68.8% of clinicians endorsing trauma‐focused interventions as safe, mental health risks to clients were described as a key barrier to treatment. Further exploration is required to unpack such apparent contradictions within complex care planning for people with psychosis.

To date, no research has examined the perspectives of UK health care professionals in this area. In the United Kingdom, mental health care (including psychological therapies) for people with a psychotic disorder is typically delivered by multi‐disciplinary community mental health teams within the publicly funded National Health Service (NHS). Clinical decision‐making is informed by clinical guidance published by the NICE and implemented into local services by clinical commissioning groups (NICE, 2014). Specialist service provision may also include early intervention in psychosis services (Lester et al., 2009) following the first episode of psychosis; inpatient and community crisis resolution teams during acute episodes of illness or risk; asssertive outreach teams (Wright et al., 2003); and longer‐term rehabilitation services (Killaspy et al., 2013).

Treatment decisions are complex, influenced by clients, clinicians, organizational, and treatment variables. The perspectives of health care professionals offer valuable insights into understanding the delivery of trauma‐focused interventions in this population and may offer valuable insights to implementation efforts. In the current study, we sought to explore and better understand health care professionals’ perceptions of the barriers and facilitators to using trauma‐focused interventions for people with psychosis and PTSD.

## METHODS

### Design

We conducted individual, semi‐structured qualitative interviews with health care professionals working in the commissioning and delivery of clinical services for people experiencing psychosis. Treatment decisions within clinical contexts are social processes involving multiple stakeholders with differing experiences and perspectives. We therefore adopted a constructivist grounded theory approach to the research design.

### Grounded theory

Grounded theory is an inductive methodology with systematic guidelines for collecting and analysing qualitative data (Glaser & Strauss, 1967). The methodology seeks to inductively discover and abstract theoretical models from data through iterative processes of data collection and analysis. Within constructivist grounded theory (Charmaz, 2014), the position and perspective of researchers are recognized as playing an active role in the construction of theory from data.

Constructivist grounded theory methods (Charmaz, 2014) begin with purposive sampling of participants with varied perspectives on the research topic. Data collection and analysis occur iteratively and coding progresses through the constant comparison of similarities and differences within the data.

### Procedure

#### Participant recruitment

Care and treatment planning within NHS clinical services for individuals with psychosis involve clinicians from several disciplines, supervisors, and managers, and decision‐making is informed by a clinical commissioning group (NICE, 2014). We aimed to capture the diverse perspectives of staff involved in this care planning process by purposively approaching clinicians from different clinical disciplines in a variety of services. Individuals working within a range of clinical services for people with psychosis across the Greater London area were contacted inviting them to participate. Participants were asked to identify colleagues with diverse perspectives, who were subsequently approached to participate.

#### Data collection

Interviews were primarily conducted face‐to‐face at the participant's place of work or at University College London, while a minority were completed via telephone. Consent forms were completed prior to face‐to‐face interviews and completed digitally prior to telephone interviews. All interviews were audio‐recorded using a digital voice recorder. Participants provided a limited amount of socio‐demographic information as well as their role and work setting at the beginning of the interview.

During the interviews, we aimed to first elicit participants’ knowledge about trauma‐focused interventions and then explore their experiences of these interventions and the barriers and facilitators to providing them. The interview schedule was developed through discussion between the research team and familiarization with existing relevant literature. The schedule was used as a prompt while the interviewer prioritized exploration of participants’ experiences. The interview schedule evolved during the data collection process, as is consistent with grounded theory methodology (Charmaz, 2014), to explore emerging themes and potential barriers and facilitators identified in earlier interviews more deeply. Following transcription of the first five interviews, we adapted the interview schedule to include a definition of trauma‐focused interventions. This was intended to clarify that the interview was primarily focused on trauma‐focused psychological interventions for PTSD, rather than trauma‐informed care more broadly (See Appendix S1).

#### Data analysis

We transcribed interviews using Express Scribe Pro software, and analysis was facilitated by NVivo 11 software. Memo‐writing and purposive sampling allowed emerging themes and concepts to be further explored in later interviews.

We initially selected a cross‐section of five contrasting interviews for line‐by‐line coding with descriptive labels. Codes were then compared to identify frequent, common, and contrasting ideas: this process generated categories or focused codes and the development of an initial coding framework.

We coded the remaining interviews using the initial coding framework. Novel codes were incorporated into the analysis as they were developed so that open and focused coding continued simultaneously. We used memos to define, refine, and elaborate coding decisions and to provide rationale and definitions of higher‐order coding.

### Quality assurance

We have endeavoured to maximize the credibility of the analysis through transparent reporting of the research processes, researcher reflexivity including a statement of the researcher's position, and the use of validity checks.

#### Reflexivity

In recognition of the role of the researchers’ perspectives in the research process (Charmaz, 2014), the researcher responsible for data collection and analysis (EC) maintained a reflective log throughout the conduct of the study. Initial reflections regarding expectations and beliefs about the research subject have been summarized into a statement of the researchers’ positions below: we hope this offers readers the opportunity to consider how our positions may influence the analysis (Elliott et al., 1999).

#### Researchers’ positions

EC: I am fortunate to have no personal lived experiences of either PTSD or psychosis. While working with adults experiencing psychosis as a research assistant, I became aware of the significant histories of the trauma of the individuals I worked with. Prior to conducting the interviews and analysis, I had limited experience delivering trauma‐focused interventions and no experience of working in NHS services for, or delivering trauma‐focused interventions to, people experiencing psychosis. This naivety about delivering trauma‐focused interventions may have limited my pre‐conceptions regarding the research question and facilitated genuine curiosity about the topic. However, it may also have limited my awareness of subtle nuances within participants’ accounts. This research was completed in partial fulfilment for the Doctorate in Clinical Psychology.

JB: I have worked with people with PTSD and psychosis for more than 20 years during my career as a Clinical Psychologist. As such I brought an ‘insider perspective’ to this research topic. This complemented the role of the first researcher and enabled us to balance curiosity and experience, while staying close to the data.

#### Validity checks

The coding and model development were led by the first author (EC). Two transcripts were independently reviewed by JB who contributed potential codes which were then compared with those developed by EC and incorporated into the provisional coding framework. The development of more focused codes and the conceptual representation of the data was then led by EC and reviewed through regular discussion in supervision to ensure credibility and face validity of the emerging framework. JB provided consultation and supervision throughout all stages of the research process.

### Ethical considerations

The study was approved by the University College London Research Ethics Committee, reference 15035/001.

## RESULTS

### Participants

We interviewed 18 health care professionals. Interviews ranged from 25 to 57 min. Participants’ ages ranged from 33 to 58 years with between 3 and 34 years of experience working in mental health services. The professions of staff were: 11 clinical psychologists, three psychiatrists, two social workers, one nurse, and one occupational therapist. Participants were working in a range of clinical teams: four in inpatient settings; three in early intervention for psychosis services; two in recovery and rehabilitation; one each in community mental health and a specialist psychological therapies service. Six participants were in senior positions across clinical teams and one in a Clinical Commissioning Group.

### A conceptual representation of results

**FIGURE 1:**
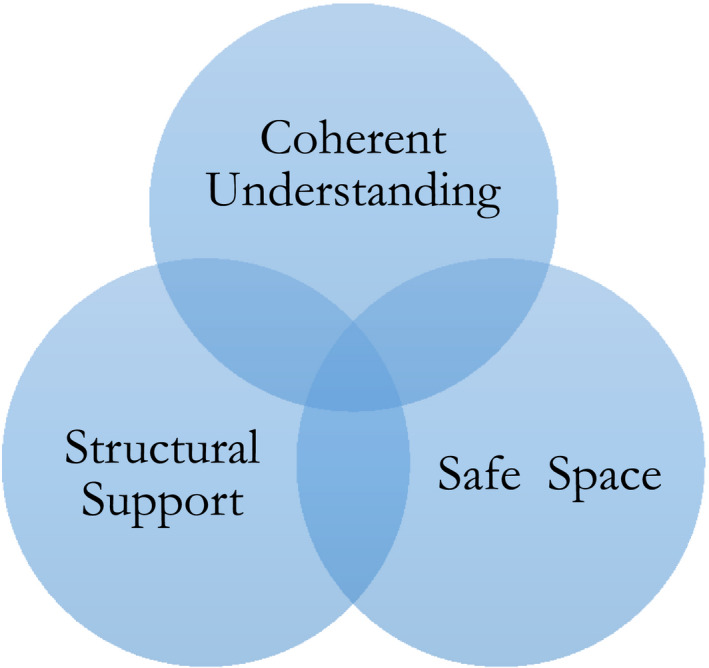
Dominant themes influencing the use of Trauma‐Focused Interventions in Psychosis

Across participants, strong shared narratives were identified regarding the barriers and facilitators to delivering trauma‐focused interventions for people with psychosis. The conceptual model comprises three dominant but overlapping themes: (i) coherent understanding; (ii) structural support; and (iii) safe space (Figure 1).

All participants recognized the prevalence of traumatic experiences in people with psychosis and their potential consequences, including PTSD. Despite this, they acknowledged that traumatic experiences and PTSD symptoms were frequently neglected in assessment and treatment planning. In the context of complex clinical presentations and symptoms, identification of traumatic experiences was positioned as a key facilitator to the recognition of symptoms as trauma‐related and subsequent access to trauma‐focused interventions.

### Coherent understanding

Participants described difficulty integrating discussion of trauma into a clinical discourse within services for psychosis. This prevented knowledge about the prevalence of trauma and trauma‐related symptoms in psychosis being translated into clinical practice including routine identification of traumatic experiences and individuals who would benefit from trauma‐focused interventions. The coherent understanding was influenced by a dominant bio‐medical model; awareness and perceptions of psychological interventions; and clinician characteristics. Illustrative quotations are included in Table 1.

**TABLE 1:** Sub‐categories and illustrative quotes of Theme 1: A Coherent Understanding

Sub‐category	Illustrative quotes
Trauma and the dominant bio‐medical model	‘the research on trauma and psychosis is a difficult one for those who believe that psychosis is an organic, degenerative brain disease’ (P11, Psychologist) ‘it's looking at the social model when we're in a medical team’ (P17, Social Worker) ‘psychiatric professionals were always quite reluctant to acknowledge trauma and that kind of drive of having trauma introduced into the, the debate very often regularly came from outside’ (P4, Psychiatrist) ‘there's rarely, only in a minority of cases, any evidence that people have been offered, um, er, a comprehensive trauma screening so that they've really been asked, in the standardised way, about um, their experience of kind of common traumatic events’ (P16, Psychologist) ‘a client's narrative may be shaped, very well shaped by what clinician's routinely ask them, so they get into a narrative of talking about psychosis‐related symptoms rather than talking about their trauma ‘cause they assume that's not what they're here for, that's not what's available, that's not what can be treated’ (P2, Psychologist)
Awareness of and perceptions about psychological interventions	‘it means different things to different people and I think that does cause um, some confusion sometimes, the lack of clarity’ (P16, Psychologist) ‘I think the psychodynamic therapy offers someone to talk about their losses and their traumas and the difficulties from their past and it can be quite deep work’ (P17, Social Worker) ‘everyone should be offered um, psychological assessment and CBT for p, um, it's the, is the stated intervention’ (P3, Occupational Therapist) ‘I don't sense here that there's any, sort of, deep work here, with the CBT’ (P17, Social Worker) ‘I ask about trauma and I can see that it can affect mental state, but actually what I can do’ (P15, Psychiatrist) ‘I think pretty much everywhere now you have to have a discrete, you offer people discrete therapy contracts that are far too short for what they actually need because that's the NHS context’ (P1, Psychologist)
Clinician characteristics	‘you need a lot of compassion, but you need compassion in such a way that you can also work out when you've got compassion fatigue and that you're burning out’ (P1, Psychologist) ‘there are some people who within the team, who just have um, more of an acute sensitivity to people's experiences and some who don't, some who are able to um, ask enough, and not necessarily, over and unpack at an assessment point um, and um, some who don't’ (P3, Occupational Therapist) ‘because I have a very intensely, intense psychodynamic background, I’m not very much in favour of this’ (P5, Psychologist)

#### Trauma and the dominant bio‐medical model

Participants described the clinical discourse within services as being dominated by a bio‐medical model of psychosis as a biological illness: this was positioned as inconsistent with discussions of symptoms in relation to traumatic experiences. While the participants spoke about biopsychosocial approaches, the bio‐medical model was described as dominating the clinical discourse within services.

the overwhelming presence of the medical model, the overwhelming presence and the overwhelming faith to chemistry (P5, Psychologist)

Participants emphasized the importance of routine questions about traumatic experiences during assessments to formulate symptoms as trauma‐related and identify individuals who may benefit from trauma‐focused interventions. However, within the medically dominated understanding, participants perceived that such questions were viewed by clinicians as a tick‐box exercise or not asked at all. This could increase the risk that PTSD symptoms were not identified nor appropriate intervention offered.

Participants also reflected on how this clinical discourse influenced service users’ own narratives about their mental health. The absence of questions about trauma, or failure to follow up disclosures, fostered an understanding that these experiences were not relevant: this could discourage service users from discussing them and further reduce the likelihood of symptoms being recognized and an appropriate intervention offered.

… talking about psychosis‐related symptoms rather than talking about their trauma ‘cause they assume that’s not what they’re here for, that’s not what’s available, that’s not what can be treated (P2, Psychologist)

#### Awareness of and perceptions about psychological interventions

Significant differences between participants’ awareness of and perceptions about psychological interventions indicated ambiguity about the nature and use of interventions. Participants described diverse definitions of trauma‐focused interventions. For some, they were conceptualized specifically as interventions involving the re‐living or re‐processing of traumatic memories. However, others described a broader approach whereby traumatic experiences were included within the clinical formulation but were not the focus of treatment. Terms such as ‘trauma‐focused interventions’ and ‘trauma‐informed approaches’ were used interchangeably. Systemic therapies, psychodynamic psychotherapy, peer support, and medication were also described as trauma‐focused interventions. This conflation of terminology highlighted ambiguity in the discussion of trauma and trauma‐focused interventions.

Participants expressed beliefs that the provision of psychological therapies, and of trauma‐focused interventions specifically, was extremely limited and difficult to access. One psychiatrist described frustration at identifying PTSD but being unable to offer treatment within the service. Provision was perceived to be limited by the number of therapists, range of available interventions, and pressures to deliver brief interventions. Overall, psychological therapy provision was characterized as inadequate to meet the complex needs of this population.

Participants also spoke about widespread narratives that talking about traumatic experiences would exacerbate distress. As such, participants acknowledged that clinicians and service users may view the re‐living of traumatic experiences as counter‐intuitive. Psychologists perceived the avoidance of talking about trauma due to the fear of exacerbating distress as a barrier to both the recognition of PTSD symptoms and engagement (of clinicians and service users alike) with trauma‐focused interventions.

it's something that is counter‐intuitive isn’t it, that…that we are asking people to believe that you get better by diving into the depths of the worst thing that ever happened to you (P13, Psychologist)

Psychologists described the importance of psycho‐education in promoting understanding and engagement in trauma‐focused treatments. Staff reflected that their own understanding of and confidence in the treatment had improved through training and clinical experience of interventions.

#### Clinician characteristics

Participants perceived clinicians’ characteristics to influence how valuable clinicians considered trauma‐focused interventions to be, as well as how sensitively and effectively they were able to talk about traumatic experiences and PTSD symptoms. Individuals reflected on how their own experiences, including traumatic experiences, influenced them to pay more attention to trauma and PTSD symptoms in their clinical practice. Personal experiences of trauma were also considered to contribute to clinician aptitude for talking sensitively about the traumatic experiences of service users. In contrast, one participant highlighted how clinicians’ personal experiences of trauma could make discussing traumatic experiences with service users feel more difficult or potentially re‐traumatizing:

many professionals have traumatic experiences, and actually are as vulnerable as clients are to re‐activating traumatic experiences (P4, Psychiatrist)

Personal qualities of the clinician were considered by psychologists to be as important as clinical skills and techniques to deliver trauma‐focused interventions. Participants specifically noted the importance of courage and compassion to enable clinicians to work with emotionally evocative content of trauma‐focused interventions.

Psychotherapeutic modality or theory was also presented as an important influence on the use of trauma‐focused interventions. Psychologists working within a systemic framework reported focussing on indirect and system‐level working over individual clinical work. Trauma‐focused interventions were described as incongruent with psychodynamic training and practice.

### Structural support

Significant structural factors, including service configuration, communication between stakeholders, and training, influenced the use of trauma‐focused interventions. Recognition of symptoms as trauma‐related and access to trauma‐focused interventions was further considered to be influenced by the culture and operational mechanisms that facilitated disclosure of traumatic experiences by service users: this was created and influenced at multiple levels including socio‐cultural context, organizational structures, and individual clinicians. Illustrative quotations are included in Table 2.

**TABLE 2:** Sub‐categories and illustrative quotes of Theme 2: Structural Support

Sub‐category	Illustrative quotes
Service configuration	‘I think it's a very traumatic experience to have touched on the emotional struggles that you have, and then, you're told ‘you're not for us though, you're not the right kind of distress, we don't do that sort of distress’’ (P3, Occupational Therapist) ‘you can then kind of get into a situation of playing bat and ball with another service’ (P2, Psychologist) ‘I think it's just, probably sometimes you know, you sort of know the response you will get, the push back that you're going to get and you know, you are struggling sometimes, so, I think that was the main factor really, in terms of preventing, me from referring’ (P7, Nurse)
Communication	‘a certain level of understanding and knowledge that they were able to […] speak the same language that the psychologist would've been speaking. So you have synergy in terms of how people would operate and intervene’ (P7, Nurse) ‘don't see the services coming back to me and saying…we need to put in place trauma‐focused therapies for psychosis in a very specific way’ (P14, Commissioner/Social Worker)
Training	‘there's probably a training need within the team, uh, around the assessment of trauma and actually understanding the impact of trauma on psychosis’ (P3, Occupational Therapist) ‘We have real problems getting specialist supervision…particularly in relation to EMDR, so I would like um, to have all the psychologists in my service training in EMDR and to have supervision, um, for delivering that with a…psychosis population. No chance.’ (P9, Psychologist)
Barriers at multiple layers	‘there is the, pervasive kind of silencing in our communities of trauma, so you know there's… there's barriers to disclosure from a service user side’ (P16, Psychologist) ‘there's so many different um, KPI’s linked with the national template, if you like, of what EIS services should provide, that it's actually quite difficult to think in the round about what really ought to be sort of more fundamental training needs within the team, um, given the amount of trauma that people experience who are on our caseload’ (P3, Occupational Therapist) ‘I looked at the…what we had in terms of psychological therapies at the time, and about 6% of our work was going on with people with psychosis, so the whole Trust had bought into this idea, this wasn't a group for whom psychology… ‘ (P11, Psychologist) ‘a high level of, of, um, need, which, um, essentially undermines our capacity to engage and maintain people within um, a psychological aspect of the pathway’ (P3, Occupational Therapist) ‘So if you've got somebody who has a special interest in trauma, maybe a national expert, you may get services that developed in a better way or a different way to services in a different area that maybe didn't have that local expertise or interest’ (P2, Psychologist) ‘when I’m training psychologists people say oh the team are very resistant, the team don't want me to do, they just wanna up the meds’ (P10, Psychologist)

#### Service configuration

Participants identified service configuration as a barrier to trauma‐focused interventions. Participants described distinct services for the treatment of PTSD and psychosis and uncertainty about which service was best placed to deliver trauma‐focused interventions for people with psychosis. This created ambiguity regarding where trauma‐focused interventions were situated within services.

I mean I do think this is a barrier, so, we…we’re a psychosis service, so in terms of what we should be offering as a service, it’s much, you know, much more it’s CBT for psychosis, as such (P8, Psychiatrist)

Referral pathways between services were experienced as challenging. Participants described variable referral outcomes and examples of referrals being declined due to differing clinical opinions; rigid, changing, and ambiguous service thresholds; and how referrals were written. Clinicians described therefore lacking confidence that referrals would be accepted and being discouraged from making future referrals.

Transitions between services could also be distressing for service users. Participants reflected that service users sometimes perceived the referral process as a rejection: their difficulties were regarded as ‘*not the right kind of distress’* (P3, Occupational Therapist). Clinicians expressed concern that the referral process could damage service user engagement with treatment and services generally.

#### Communication

Participants talked about the importance of communication between members of the multi‐disciplinary team in promoting appropriate referrals for psychological interventions. Limited communication and the use of psychological terminology appeared to create friction in communication between psychological therapists and colleagues from other disciplines. Clinicians described efforts to improve communication between colleagues from different professional disciplines through training, supervision, and a forum for team formulation.

Communication between services and with commissioners was also emphasized. Participants talked about how outcomes and feedback from referrals to other services informed future decision‐making. The frequent absence of feedback left clinicians uncertain about the appropriateness or outcomes of referrals.

We’ve referred patients there instead, but I must say the difficulty then is, the people who are accepted for treatment, I must say I don’t get much feedback (P8, Psychiatrist)

Feedback from clinical services to commissioners was also described as an important influence on service planning and development.

#### Training

Staff training on the assessment of traumatic experiences, PTSD symptoms, and delivery of trauma‐focused interventions was also discussed by participants. Participants highlighted the importance of management and organizational endorsement of training through the provision of funding and protected time for staff to attend training.

you would run a training and if it was an opt‐in option for staff, then it would very much depend on how busy staff felt they were, and if staff were over run with other things, it was, managers weren’t insisting they go, they just wouldn’t come […] it would feel like a luxury to go to training on something like that. (P2, Psychologist)

Ongoing supervision following training was considered important to support clinician confidence and competence in the assessment of PTSD and delivery of trauma‐focused interventions.

#### Barriers at multiple layers

Participants spoke about the significant barriers to service users disclosing traumatic experiences and seeking help for PTSD symptoms: these included the stigma and shame often associated with such experiences. They spoke about the need for a culture that facilitated disclosure and how this was created and influenced at multiple levels from social‐political context to individual clinicians. Efforts to facilitate access to trauma‐focused interventions, therefore, required similar attention at multiple levels.

At a socio‐political level, participants spoke directly about feminism and the ‘Me Too’ movement as increasing public awareness and normalizing the discussion of trauma. Others highlighted how poverty and austerity increased the risk of people experiencing trauma and reduced funding of health, social, and community services.

Staff within managerial roles spoke about the influence of national policy and implementation guidance on service practices. They described difficulty prioritizing service development work around the identification and treatment of PTSD in the context of extensive national‐level key performance indicators.

At an organizational level, participants highlighted the role of senior leadership in allocating resources and training to the assessment of PTSD and delivery of trauma‐focused interventions within psychosis services. Mechanisms such as patient record systems and the availability of screening tools also impacted clinical practice. Senior leaders were therefore perceived as influential in creating the organizational culture and processes that facilitated disclosure of trauma and delivery of trauma‐focused interventions.

Individual therapists described feeling supported or inhibited in delivering trauma‐focused interventions by the culture and attitudes held by colleagues and the wider team. Sustaining practices designed to improve the routine identification of traumatic experiences and delivery of trauma‐focused interventions required endorsement and structural support at multiple levels of the organizational hierarchy and broader cultural context.

### Safe space

Safety was prominent in clinicians’ minds when discussing the disclosure of traumatic experiences and delivery of trauma‐focused interventions. Participants acknowledged anxiety about the risk that assessment of traumatic experiences, PTSD symptoms, and trauma‐focused interventions could cause harm to both service users and clinicians. Finally, clinicians talked about the skills required to safely enquire about trauma and deliver trauma‐focused interventions. Illustrative quotations are included in Table 3.

**TABLE 3:** Sub‐categories and illustrative quotes of Theme 3: Safe Space

Sub‐category	Illustrative quotes
Achieving Sufficient Safety	‘I would…very much…use that principle that the processing comes when safety has been achieved’ (P12, Psychologist) ‘there are also things about the containment of the environment that actually, in some ways make it easier, so from session to session, there's more people around to help people stay safe, and support them in promoting their own safety’ (P13, Psychologist) ‘if she or he has the resource to deal with that during the, the trauma therapy’ (P15, Psychiatrist) ‘people need to first of all trust the team they work with and the professionals they work with enough […] so it's creating the safe space’ (P4, Psychiatrist) ‘it's important that she got a sense that she could trust me, and that we could work together, I thought we shouldn't start with the trauma, and that we should do some work on the social anxiety first’ (P9, Psychologist) ‘it was felt that the person would not have been able to, because of how chronic they are with their symptoms and how long‐standing their illness has been’ (P7, Nurse)
Potential for harm	‘people worry that the process of talking through the trauma will raise so much distress that people with psychosis in particular won't be able to manage that, and therefore that it will have a knock on effect on their other symptoms say’ (P10, Psychologist) ‘They're then self‐harming and you did that…that's your fault, and there's bound to be a bit of a narrative about that’ (P13, Psychologist) ‘I think there's quite, a kind of naïve understanding for some care coordinators, um, that any exp‐emotional expression is very dangerous and wrong, […] and um, you know, people should avoid talking about things that upset them’ (P9, Psychologist) ‘you've got a staff group that are also terrified of, you're gonna open up a can of worms, don't go back there either’ (P18, Psychologist) ‘there's something about working with trauma…um, that is…quite hard going, um and it's quite draining and there, there is a risk of vicarious traumatisation’ (P10, Psychologist) ‘we can only help them if we're not burnt out ourselves’ (P17, Social Worker)
Clinician skills	‘we don't have the skills to contain, to handle, to respond safely, to a patient perhaps telling us something’ (P17, Social Worker) ‘when we've actually asked staff they've just said well yeah, we, we, we feel like we don't know how to do this’ (P16, Psychologist) ‘they may not therefore have the training, have the supervision, have the know‐how how to do it, and…and therefore you know, none of us are gonna be doing work if we, or we shouldn't be doing work if we're not competent to be doing it, it's important that we have those competencies to be doing the work’ (P10, Psychologist)

#### Achieving sufficient safety

Clinicians described *‘creating the safe space’ (P4*, *Psychiatrist)* to ask about trauma or start trauma‐focused interventions. When speaking about the requirement of sufficient safety, participants spoke about a range of conditions including the timing of treatment, affective regulation, and coping strategies, as well as the acuteness or chronicity of their symptoms or clinical presentation. They also emphasized the importance of a supportive environment including social networks; community and clinical resources and the relational safety of the therapeutic relationship. The delivery of trauma‐focused interventions was therefore predicated on the clinical assessment of sufficient safety.

Participants described strategies to address tractable barriers including establishing greater safety and stability before embarking on re‐processing work. Psychologists described a stabilization phase of treatment involving the development of skills such as emotional regulation, grounding, self‐soothing, and mindfulness. Other clinicians emphasized engagement and developing a therapeutic relationship to establish relational safety. Finally, therapists described focusing on other clinical targets, such as social anxiety or hearing voices, before commencing trauma‐focused interventions.

Participants acknowledged that they did not always feel able to achieve sufficient safety. Perceived limitations to community and clinical service provision contributed to concerns that services were not able to provide a context of sufficient safety for trauma‐focused interventions. Participants also acknowledged that relational safety could be challenging where services themselves had been experienced as traumatic and harmful.

people might struggle more to accept a service from a service which has already traumatised them at their point of entry (P3, Occupational Therapist)

#### Potential harm

Anxieties that trauma‐focused interventions could cause harm were raised by participants. While participants acknowledged service user anxieties, the anxiety experienced by clinicians and clinical teams was more prominent within discussions.

Participants described both their own and colleagues’ anxieties that trauma‐focused interventions could be destabilizing and increase service user distress. At its most severe, clinicians expressed concern about the exacerbation of symptoms of psychosis and risk of potential adverse events including suicide. These fears appeared to be intensified by incongruence between trauma‐focused interventions and clinicians’ conceptualization of mental health treatment (Theme 1).

Anxieties about the risk of causing harm were confounded by the potential harm to clinicians themselves. Participants talked about the emotional impact on therapists of delivering trauma‐focused interventions, and about the possibility of vicarious traumatization. Psychologists described potentially catastrophic professional and reputational consequences for therapists if service users’ mental health deteriorated or they attempted suicide during treatment:

real consequences for that person’s career […] perhaps they'll be viewed as negligent and perhaps it’ll impact whether they can continue to practice (P1, Psychologist)

While psychologists acknowledged experiencing anxieties about trauma‐focused interventions, few reported that these worries prevented them from delivering trauma‐focused interventions. Participants described research evidence and personal clinical experience on the effectiveness of trauma‐focused interventions as outweighing anxieties about treatment. Support from other psychologists and colleagues from other disciplines, supervisors, and managers, was identified as enabling the delivery of trauma‐focused interventions despite this anxiety.

#### Clinician skills

Clinicians’ skills were an important facilitator to the identification of traumatic experiences and assessment of PTSD symptoms. Participants described not believing they had the skills to respond to disclosures of traumatic experiences in a safe, helpful and therapeutic manner. Such clinician self‐evaluations of their clinical skills could inhibit them from inquiring about traumatic experiences and PTSD.

Psychologists described questioning their own competence to deliver trauma‐focused interventions, as well as the competence of clinicians specializing in trauma‐focused interventions to work with individuals with psychosis. While some described developing greater confidence over time, others remained uncertain of their competencies. Clinicians’ lack of confidence either in their own competence to deliver trauma‐focused interventions or the competence of others working with people experiencing psychosis may prevent service users from being offered intervention in either clinical context.

he sits within a psychosis service, so there is always a slight kind of concern that maybe my competencies aren't there (P2, Psychologist)

## DISCUSSION

This study captured the perspectives of 18 health care professionals and offers novel insights into the barriers to the delivery of trauma‐focused interventions for people with psychosis and PTSD. Identified barriers were similar to those reported by clinicians in treating PTSD more generally (Finch et al., 2020), although some were also unique to the population of people with psychosis and PTSD. Our findings are consistent with and offer added confidence to previously reported barriers to trauma‐focused interventions in people with Severe Mental Illness (Salyers et al., 2004) and first‐episode psychosis (Gairns et al., 2015). The findings extend the existing literature by offering a novel and rich exploration of three conceptualized themes: coherent understanding; structural support; and safe space, through a number of sub‐themes.

Participants perceived difficulty coherently integrating trauma‐focused interventions into the clinical discourse of services for psychosis: explanations for this included the dominance of a bio‐medical model of illness, understanding of psychological therapies, and clinician characteristics. While the tensions in the implementation of the biopsychosocial model have been discussed extensively elsewhere (Alvarez et al., 2012; Papadimitriou, 2017), this study is the first to explore this tension in relation to the delivery of trauma‐focused interventions for people with psychosis, and was not a barrier identified in the treatment of people with PTSD without comorbid psychosis (Finch et al., 2020). These perceived difficulties coherently integrating clinical discourses of psychosis and trauma may offer possible explanations of previous findings that clinician knowledge about trauma in this population was not predictive of clinical practice (Salyers et al., 2004).

In line with previous research (Finch et al., 2020; Gairns et al., 2015; Salyers et al., 2004), health care professionals emphasized organizational influences on the delivery of trauma‐focused interventions. Participants were motivated to integrate trauma‐focused interventions into their clinical work with people with psychosis. However, in the absence of the organizational culture and structural support such as training and clear referral pathways, they did not consider these routine practice. The findings show multiple layers of organizational context as actively influencing the delivery of trauma‐focused interventions, and challenges the notion of an organization as the passive environment in which implementation of interventions occurs (Nilsen, 2015).

Health care professionals emphasized the need for a safe space to deliver trauma‐focused interventions. Clinician anxieties about the potential mental health risks of trauma‐focused interventions have been previously documented in the treatment of PTSD (Becker et al., 2004; Finch et al., 2020) and Severe Mental Illness (Salyers et al., 2004). The current analysis additionally highlights clinicians’ anxieties about the possible emotional, reputational, and professional consequences to clinicians delivering interventions. Clinical supervision has been associated with reductions in therapist‐reported harm expectancies regarding trauma‐focused interventions (van den Berg, de Bont, et al., 2016; van den Berg, van der Vleugel, et al., 2016). Current findings indicate those clinician anxieties can be mediated by a range of sources of support beyond clinical supervision including relationships with colleagues from multiple professional disciplines, management attitudes, and organizational culture.

While participants acknowledged the role of client‐related barriers to trauma‐focused interventions, they were not conceptualized as a dominant barrier as reported in other studies (Salyers et al., 2004; Frueh et al., 2006; Gairns et al., 2015). This is consistent with Finch et al. (2020) systematic review of the barriers and facilitators to the implementation of trauma‐focused interventions for people with PTSD more generally, where the authors noted that the reporting of client‐related factors was limited across all 34 included studies. This may be due to client‐related factors being perceived as a less important determinant of access to trauma‐focused interventions, or maybe as a result of this being examined less in research, and warrants further exploration.

Exposure to traumatic experiences is common and most who experience traumatic events do not go on to develop PTSD (Breslau, 2009). However, we note that participants often prioritized the identification of traumatic experiences, rather than symptoms of PTSD. In complex clinical presentations, symptoms of PTSD may be ambiguous, under‐estimated, or overlap with symptoms of psychosis (Dallel et al., 2018; O’Conghaile & DeLisi, 2015). Awareness of traumatic events may function to screen for and formulate symptoms as trauma‐related that may otherwise be overshadowed or attributed to psychosis. However, PTSD is under‐recognized in this population even when traumatic experiences are recorded in clinical notes (de Bont et al., 2015). This dissonance between health care professionals’ perspectives and clinical data warrants further exploration. Participants also tended to conflate specific ‘trauma‐focused interventions’ with ‘trauma‐informed care’ more broadly. This further highlights the need to improve awareness and detection of PTSD specifically to facilitate access to appropriate evidence‐based treatment.

### Limitations

Our analysis is grounded in the perspectives of the participant sample and research team. The sample included limited numbers of health care professionals working in commissioning and from disciplines including nursing and social work who typically occupy care co‐ordinating roles within clinical teams. While we sought to include diverse views, most participants expressed positive attitudes towards treatment and negative attitudes were limited. This may be partially accounted for by the self‐selecting participant sample and nature of the research question. While the analysis generated a rich and broad exploration of themes, other individuals may have introduced different views to those presented.

Participants were health care professionals and were not asked to comment on the perspectives of people with psychosis. The voice of those with lived experience of psychosis is therefore absent from the analysis. Findings represent a partial understanding of barriers to trauma‐focused interventions: one which may emphasize organizational barriers and minimize clinician‐related barriers. Exploration of the perspectives of experts by experience would offer an additional dimension to understanding the barriers to treatment.

We adapted the interview schedule during the research process to include a standardized definition of trauma‐focused interventions. This was following our observation that participants tended to talk about ‘trauma‐focused interventions’ and ‘trauma‐informed care’ interchangeably. This addition to the interview schedule was intended to clarify that the research was focused specifically on trauma‐focused interventions. We hoped this revision would increase the clarity and consistency of the terminology used. It however highlights the frequent ambiguity in language and conflation of terms in the area of psychological trauma, which impedes clarity in both clinical and academic discussion.

### Clinical and research implications

Health care professionals described difficulty coherently integrating knowledge about the prevalence of trauma and PTSD in this population into clinical discourse and decision making: efforts to address this may occur at both local and national levels. Locally, participants spoke directly to the value of regular psychosocial team formulation sessions, which have previously been linked to the development of coherent and multi‐disciplinary care plans in early intervention for psychosis teams (Cairns et al., 2015). These may offer particular added value to the understanding of complex clinical presentations such as comorbid PTSD and psychosis. National directives such as the development of trauma‐informed services (NHS England, 2019) and the National Trauma Training Programme (NHS Education for Scotland, 2017) may facilitate the routine discussion of trauma within clinical services, thereby supporting the disclosure of traumatic experiences and identification of trauma‐related difficulties in people with psychosis. However, greater attention still needs to be paid to identifying PTSD symptoms specifically.

Health care professionals emphasized the role of structural support in providing the processes and pathways for assessment and treatment of trauma‐related symptoms in routine clinical practice. Organization‐level interventions may offer scaffolding to facilitate routine identification of PTSD and delivery of trauma‐focused interventions. Screening tools including the Trauma and Life Events checklist (TALE; Carr et al., 2018) and Trauma Screening Questionnaire (Brewin et al., 2002) may facilitate routine screening for and increase identification of trauma‐related symptoms in this population (de Bont et al., 2015). Provision of service policies, standardized processes and service pathways may ensure clarity and increase access to trauma‐focused interventions.

Staff anxieties regarding their skills in the assessment of traumatic experiences, PTSD, and delivery of interventions indicate training may play an important role in facilitating the delivery of trauma‐focused interventions. Effective training programmes may complement theoretical learning with technical skill development and ongoing clinical supervision to promote staff confidence and reduce therapist harm expectancies (van den Berg, de Bont, et al., 2016; van den Berg, van der Vleugel, et al., 2016).

The proposed model offers a valuable first step in understanding the barriers to delivering trauma‐focused interventions to people with psychosis. The applicability of this model within a broader sample could be evaluated through the assessment of a questionnaire grounded in the identified themes. The analysis could further examine the relative contribution of each in clinical decision making, to inform and tailor efforts to maximize the implementation of trauma‐focused interventions in this population.

## CONCLUSION

This study offers a rich exploration of the experiences of health care professionals in delivering trauma‐focused interventions to people with psychosis. Within a diverse sample, there existed significant common ground regarding widespread and numerous barriers to treatment. As a result, the delivery of trauma‐focused interventions was considered the exception rather than the rule. The presented themes of coherent understanding, structural support, and safe space offer both insights into the current barriers to treatment, as well as potential opportunities to address these to improve implementation efforts.

## CONFLICTS OF INTEREST

All authors declare no conflict of interest.

## AUTHOR CONTRIBUTION

**Eleanor Chadwick:** Conceptualization (equal); Formal analysis (equal); Investigation (equal); Methodology (equal); Project administration (equal); Validation (equal); Visualization (equal); Writing – original draft (equal); Writing – review & editing (equal). **Jo Billings:** Conceptualization (equal); Investigation (equal); Methodology (equal); Supervision (equal); Validation (equal); Visualization (equal); Writing – review & editing (equal).

## Data Availability

The data that support the findings of this study are available from the corresponding author upon reasonable request. Research data are not made widely available due to the potentially sensitive and personal nature of participants’ views.
