# Supplementary material for: Barriers to delivering trauma‐focused interventions for people with psychosis and post‐traumatic stress disorder: A qualitative study of health care professionals’ views
Source: Psychol Psychother. 2022 Feb 5;95(2):541–60. doi: 10.1111/papt.12387 (PMC9304310; doi:10.1111/papt.12387)
Supplement: Supplementary file 1 [file PAPT-95-541-s001.docx]

**
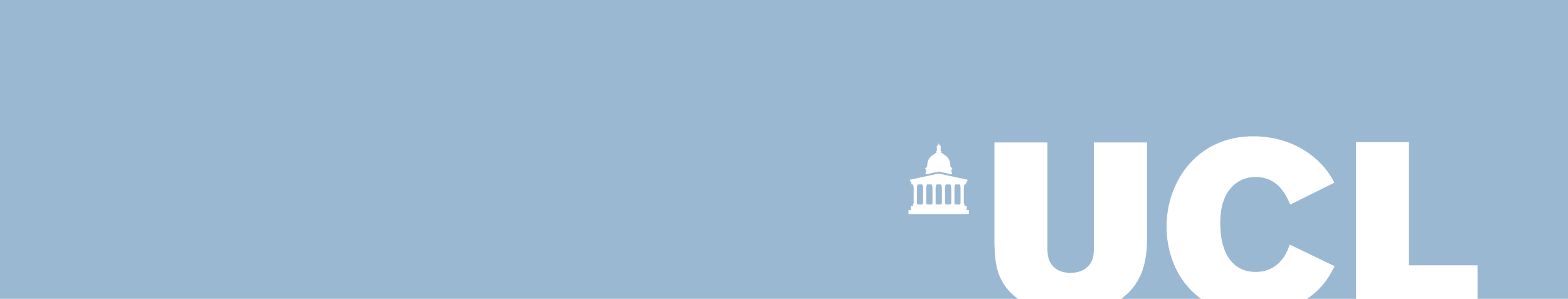
**

Interview Schedule

**LONDON’S GLOBAL UNIVERSITY**

**UCL DIVISION OF PSYCHOLOGY AND LANGUAGE SCIENCES**

Thank you for agreeing to meet with me today to talk about ‘trauma-focussed interventions in psychosis’. Maybe I could start by asking what you understand is meant by the term ‘trauma-focused interventions in psychosis’?

For the purposes of these interviews, we’re defining trauma-focused interventions as psychological interventions which seek to treat symptoms of PTSD. These might include for example, tfCBT or EMDR.

In your experience, what factors influence whether you (or your service) adopt these interventions with clients?

I wonder if you can give me an example of where you or your service have used these interventions:

What influenced your decision to use this interventions?

What made it possible to do this?

Were there any barriers/issues which arose in trying to do this?

Are there times when you/your service have chosen not to use these interventions?

What influenced your decision?

Were there any barriers/issues which stopped you from using these interventions?

Are there any considerations that make you more or less likely to adopt these interventions in your work?

Possible additional prompts (areas identified in previous research)

- Are there any aspects about a **client** that would influence whether you use this type of intervention with them?
- In your experience, do you feel that other **clinicians** are more/less likely to use this type of intervention? Why do you think that might be?
- Is there anything about the level of team, service, trust, national health service or wider socio-political level that feeds into these decisions?
- Are there any factors about the **treatment** itself that influence whether you adopt this type of intervention with clients?

NB: The research adopts a Grounded Theory methodology: as a result, this interview schedule is intended to provide initial prompts, but the interviewer will prioritise and explore insights provided by participants.
